# PEGylated lipids in lipid nanoparticle delivery dynamics and therapeutic innovation

**DOI:** 10.3762/bjnano.16.133

**Published:** 2025-10-30

**Authors:** Peiyang Gao

**Affiliations:** 1 Independent researcher, 140 First St, Cambridge, MA, 02140, USA

**Keywords:** functionalized PEG, immunogenicity, lipid nanoparticles, PEG alternatives, PEG lipids, therapeutic delivery

## Abstract

Lipid nanoparticles (LNPs) have become significant vehicles in the delivery of therapeutic substances, particularly for nucleic acid vaccines and gene therapies. A key component in the nanoparticle formulation is polyethylene glycol-modified (i.e., PEGylated) lipids (PEG lipids), which can significantly influence the stability, cell interactions, and overall effectiveness of LNP delivery vehicles. This review collates insights into the role of PEG lipids in LNPs by illustrating how the PEG chains arrange on the nanoparticle surface and the potential impacts on LNPs’ physicochemical properties by varying surface PEG density or PEG chemistry. Subsequently, PEG conformations are discussed in terms of their modulation of protein corona formation, cellular uptake, and immunogenic responses, particularly the pathways of anti-PEG antibody production and complement activation. Building on these understandings, functionalized PEG lipids are reviewed for ligand conjugation and targeted LNP delivery function. Promising alternatives to replace the benchmark PEG lipids are also systematically reviewed to address PEGylation associated immunogenicity. By conducting a critical analysis of the recent literature and identifying potent candidates for PEGylation strategies or PEG-free platforms, this review aims to provide insights and support the advancement of LNP mediated delivery.

## Review

### Introduction

Lipid nanoparticles (LNPs) have become a promising platform in modern nanomedicine, especially for delivering genetic payloads such as mRNA and siRNA. These nanoscale particles can encapsulate and protect a variety of drug substances, thereby enhancing their stability and therapeutic efficacy within biological systems [1]. LNPs are currently composed of ionizable lipids, helper lipids, sterols, and polyethylene glycol-modified (i.e., PEGylated) lipids (PEG lipids); each of these components plays a specific role in maintaining the nanoparticles’ structure and enhancing their performance [2]. The remarkable success of mRNA-based COVID-19 vaccines has highlighted LNPs as a transformative nanomedicine, driving significant interest and innovation in this field [3].

As a key ingredient in LNPs, PEG lipids are widely used to provide the nanoparticles with a unique outer layer. The “stealth” properties of PEG chains can prevent nanoparticle aggregation, reduce nonspecific protein adsorption, and delay immune recognition, thereby extending LNP circulation half-life in the bloodstream [2,4]. Because of these benefits, incorporating PEG lipids into LNP formulations has become a useful strategy for improving the colloidal stability of nanoparticles, facilitating their distribution throughout the body, and enhancing therapeutic effectiveness. However, this PEGylation strategy is not without challenges; for instance, it can lead to faster immune clearance upon repeated dosing [5]. These characteristics highlight the critical role of PEG lipids in achieving a balance between stability and functionality in LNP formulations [6]. To overcome persistent challenges and extend the utility of PEGylation strategies in LNP-based drug delivery systems, approaches are being explored. These include integrating functional groups into PEG lipids for ligand conjugation and improved cell-specific targeting, as well as developing PEG alternatives to mitigate anti-PEG antibody associated immunogenicity [7–8].

This review first reveals the localization and conformation of PEG lipids on the LNP surface, which is fundamental for understanding how PEG lipids contribute to nanoparticle stability and surface interactions. It then demonstrates how PEG density and chemical structure may influence the physicochemical properties of LNPs including particle size, surface charge, and encapsulation efficiency. Subsequent sections explore the roles of PEG lipids in modulating protein corona formation and cellular uptake. The latter parts highlight the potential of functionalized PEG lipids for targeted delivery and the development of promising alternatives to replace commonly used PEG lipids. By discussing the recent research findings, this review can serve as a guide to the design of surface-engineered or PEG-free LNPs to overcome the persist challenges in LNP delivery systems using conventional PEG lipids.

### Effects of PEG lipid surface density and structure on LNP physicochemical properties

Understanding the spatial organization of lipid components within LNPs is critical for optimizing their physicochemical characterization and stability. In particular, the localization of PEG lipids plays a significant role in providing steric stabilization and controlling interactions during blood circulation [9]. A study using small-angle neutron scattering (SANS) with selective deuteration has provided compelling insights into the lipid localizations. In a LNP formulation composed of MC3, DSPC, cholesterol, and DMPE-PEG2k at a molar ratio of 50:10:38.5:1.5, SANS analysis revealed a core–shell architecture with an average particle diameter of approximately 75 nm and a LNP shell thickness of 5 to 7 nm. Quantitative modeling of shell composition further demonstrated that the DMPE-PEG2k lipid accounted for 3.0 ± 0.5% of the shell volume, alongside 38 ± 7% MC3, 33 ± 3% cholesterol, and 26 ± 4% DSPC. Notably, PEG lipids were found exclusively in the shell region, with no detectable presence in the core area [10]. This localization of PEG lipids aligns with their intended function to form a sterically repulsive layer that stabilizes LNPs during formulation and minimizes nonspecific bindings during blood circulation.

In the utilization of PEG lipids to create PEGylated LNPs, it is important to consider not only where these PEG lipids reside but also how the conformations of PEG chain can influence nanoparticle behavior. Once anchored to the LNP shell, PEG chains can adopt the spatial arrangement of either “mushroom” or “brush” conformation [11]. These two different PEG chain conformations are primarily governed by the PEG lipid density on the nanoparticle surface. At lower PEG density, individual PEG chains can space far enough apart to coil freely, forming a mushroom-like structure. In contrast, a higher PEG density forces the chains to extend outward into a more linear and, thus, form a brush-like conformation. This brush conformation can provide enhanced steric stabilization to more effectively prevent nanoparticle aggregation [12].

A structural analysis of mRNA-LNPs has provided compelling evidence that the conformation of PEG lipids on the nanoparticle surface plays a significant role. LNPs composed of ionizable lipid ALC-0315, DSPC, cholesterol, and PEG lipid ALC-0159 at a molar ratio of 46.3:9.4:42.8:1.6 were analyzed by high-field ^1^H NMR spectroscopy and diffusion-filter techniques. The ALC-0159 PEG lipid consists of a PEG2k chain conjugated to a C14–C14 dialkyl glycerol anchor. The surface PEG chain density was estimated at 27.3 PEG chains per 100 nm^2^ according to the average LNP diameter of 77 nm; this exceeded the critical surface density of 11.0 chains per 100 nm^2^, which implies a brush conformation. The reported narrow NMR line widths for PEG methylene and methoxy protons reflect rapid isotropic molecular motion, which in this context indicates that the PEG chains are not entangled [11]. This degree of motional freedom could correspond to a brush conformation signature, where adjacent PEG chains force them into a linear orientation. In contrast, PEG chains in mushroom conformation would exhibit restricted motion and thus broader peaks [13].

**Figure 1:**
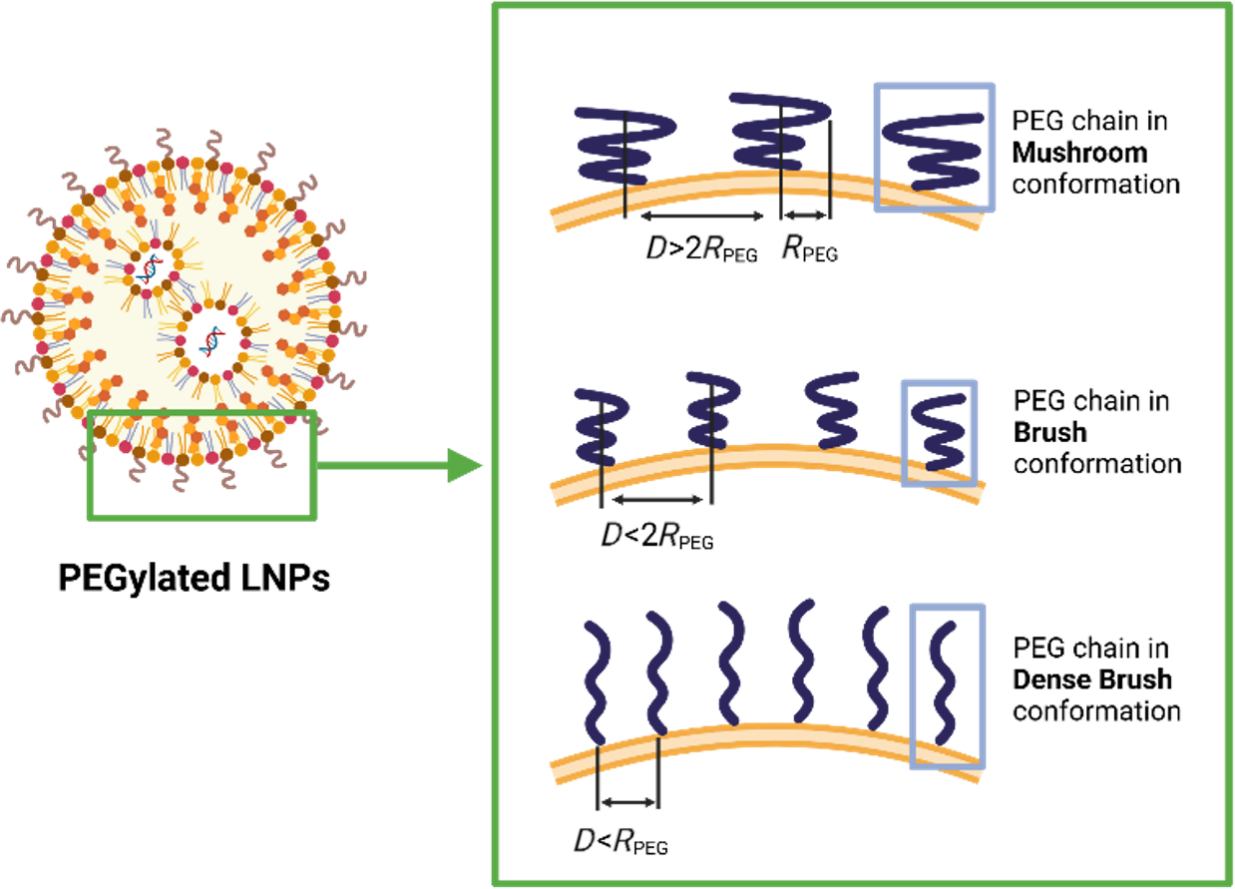
Structural conformations of PEG on lipid nanoparticle surfaces. At low PEG densities (*D* > *2R*_PEG_), the chains adopt a collapsed “mushroom” conformation that leaves parts of the LNP surface accessible to serum proteins. With increased density (*D* < 2*R*_PEG_), the PEG adopts a “brush” configuration, offering partial surface shielding while still permitting limited ligand-receptor interactions. At very high densities (*D* < *R*_PEG_), the chains pack into a dense brush layer, forming a robust steric barrier that almost completely prevents nonspecific protein binding. Figure 1 was created with BioRender. Gao, P. (2025) https://BioRender.com/mpl3g35. This content is not subject to CC BY 4.0.

While direct investigations into PEG conformations on LNPs remain limited, substantial insights can be drawn from studies on liposomes and polymeric nanoparticles. As shown in Figure 1, the PEG chain conformation is found to be fundamentally determined by the PEG grafting density on the nanoparticle surface, which can be quantitatively estimated by the Flory radius (*R*_PEG_) of PEG and the interchain distance (*D*). According to polymer scaling theory, when *D* exceeds twice *R*_F_ (*D* > 2*R*_PEG_), chains exist as isolated mushrooms with minimal overlap, enabling protein penetration to the nanoparticle surface. Experimental validation using quartz crystal microbalance with dissipation monitoring (QCM-D) reveals that reducing *D* below *R*_PEG_ (*D* < *R*_PEG_) forces chain extension through lateral compression, forming brush conformations where the excluded volume effect dominates [14]. For PEG2k (*R*_PEG2k_ ≈ 8.2 nm), achieving this brush regime requires interchain distances below 4.1 nm. Importantly, neutron reflectometry studies showed that the thickness of the hydration layer increases with the *R*_PEG_/*D* ratio, a geometric parameter now recognized as critical for predicting the in vivo fate of nanoparticles [15]. Pharmacokinetics data in the study further demonstrated that the particles with brush-like PEG conformation achieved a 19.5 h terminal half-life, versus 15.5 h for mushroom-like conformation and 0.89 h for particles without PEGylation. Particles with brush-like PEG also exhibited 1.5-fold lower clearance than the particles with mushroom-like conformation [16]. Therefore, the density-dependent conformation of PEG lipids is a critical parameter to be considered in the application of surface PEGylation strategies.

Incorporating PEG lipids into LNP formulations may also affect the physicochemical characterizations of LNPs [17]. In a recent study evaluating C12-200-based LNPs formulated with varying DMPE-PEG2k content from 1 to 5 mol %, changes were observed in nanoparticle diameter, polydispersity index (PDI), surface charge, and payload encapsulation efficiency (EE%). Increasing the PEG lipid content led to a reduction in the hydrodynamic diameter of LNPs, with particle size decreasing from 173.9 nm at 1 mol % to 109.1 nm at 5 mol %. Meanwhile, the PDI increased modestly from 0.060 to 0.146 with the increase of PEG density from 1 to 5 mol %, indicating a slight broadening in size distribution and less LNP homogeneity at higher PEG levels. The surface charge on the LNP outer shell showed a slight tendency toward more negative values as PEG density increased, from −2.84 mV at 1 mol % to −4.11 mV at 5 mol %, though the difference falls within the measurement variability. The EE% of mRNA payload remained greater than 90% across all PEG levels, though a small downward trend was noted from 98.2% to 94.8% with increased PEG molar fraction [18].

Another study has systematically screened PEG lipids across a variety of structural families including glyceride, phosphoethanolamine, cholesterol (Chol), and ceramide, to evaluate how the molecular weight and molar fraction of PEG lipids can affect LNP characterizations. In DMPE-PEG5k LNPs, increasing the PEG lipid fraction from 1.5 mol % to 5 mol % resulted in no significant change in particle size but led to a substantial encapsulation efficiency drop from over 90% to below 80%. While in LNPs incorporating Chol-PEG, higher PEG molar fraction and PEG chains with greater molecular weight both reduced particle size, with Chol-PEG5k consistently producing the LNP with smallest diameter at each PEG molar fraction tested. In Chol-PEG1k and Chol-PEG2k LNPs, the payload encapsulation efficiencies stayed within the range between 85% and 90%, even at the highest PEG molar fraction of 5%. However, Chol-PEG5k LNP has shown a notably drop in EE% from over 90% to barely 80% with the increase in PEG molar fraction, indicating that the change in EE% could depend on molecular weight [19].

Collectively, the impact of PEG lipids on LNP physicochemical properties can be affected by a complex interplay between PEG molar fraction, molecular weight, and lipid anchor structure [20]. While increasing PEG density often leads to reduced particle size, the fluctuation of size can still vary depending on the PEG anchor structure [19]. Some PEG lipids exhibit sensitivity in encapsulation efficiency at higher PEG molar fraction, while others can maintain stable payload encapsulation regardless of PEG density. Likewise, changes in size distribution and surface charge can be modest or pronounced depending on the specific PEG lipid incorporated [17–20]. These findings indicate that trends in LNP characterization change by varying PEG lipids cannot be universally applied across all PEGylated LNP formulations. Rational LNP design should consider the distinct structural attributes of PEG lipid, recognizing that optimization of LNP physicochemical properties would be PEG-specific and not translatable between studies.

### LNP pegylation-associated trade-off between nonspecific binding and cellular interactions

When LNPs are introduced into biological systems, they encounter a complex milieu of biomolecules, predominantly proteins, which can adsorb onto the nanoparticle surfaces to form a “corona” [21]. This dynamic layer, often called the biomolecular or protein corona, can significantly influence the biological identity and behavior of nanoparticles [22]. Among the factors shaping corona formation, the presence of a PEG layer on the outer shell of LNPs plays a pivotal role. As hydrophilic and protective shield during LNP circulation and in vivo activities, the conformation and binding kinetics of PEG layer are both important, particularly in modulating protein adsorption and subsequent interactions with cellular uptake pathways [23].

**Figure 2:**
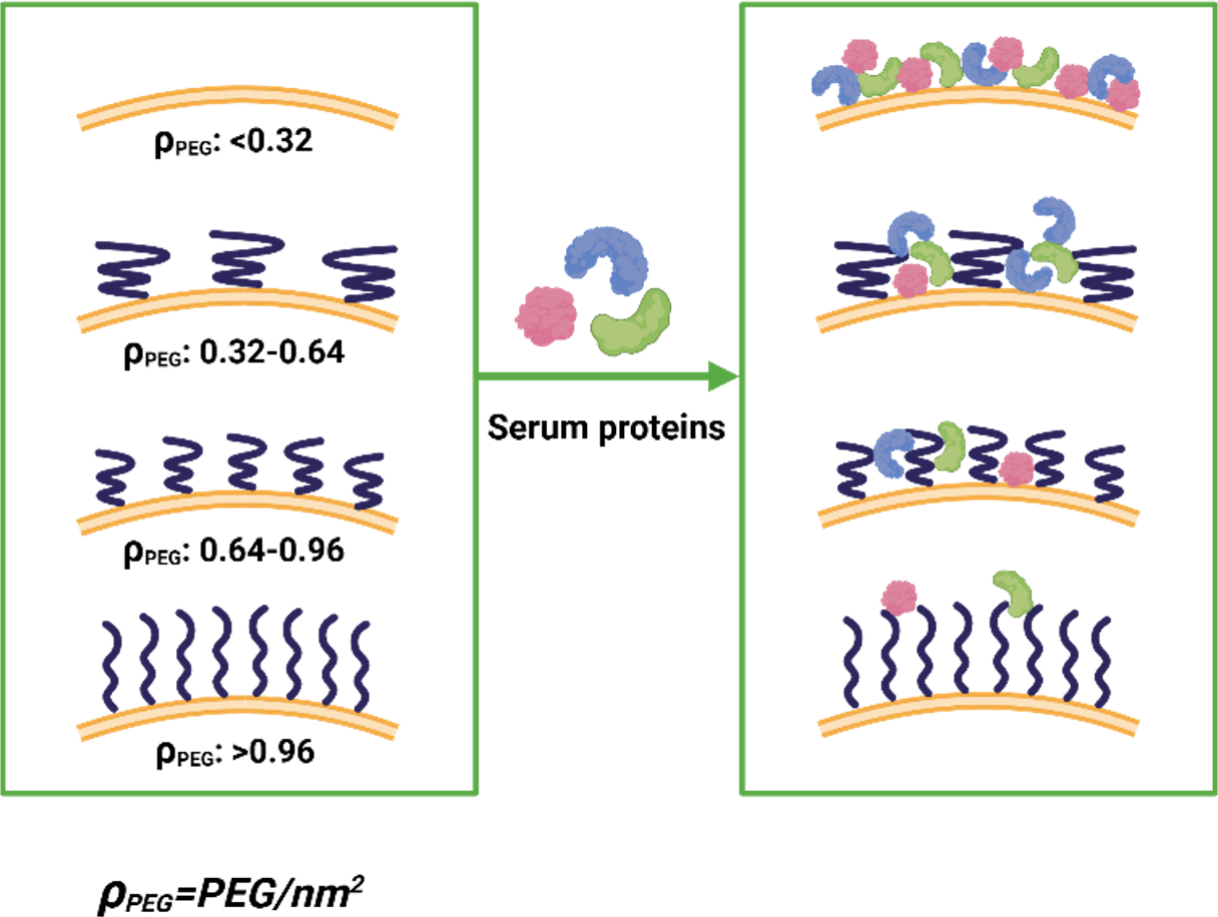
Influence of PEG surface density on serum protein binding. The PEG surface density can be quantified as the number of PEG chains per square nanometer LNP surface area (ρ_PEG_) to correlate with the serum protein adsorption rate. Sparse coverage (ρ_PEG_ < 0.32 nm^−2^) permits extensive binding of proteins such as albumin and fibrinogen, which can accelerate immune recognition and clearance. At intermediate densities (0.32 nm^−2^ < ρ_PEG_ < 0.96 nm^−2^), partial surface shielding reduces protein adsorption while still enabling some ligand accessibility. Further increased PEG density leads to additional suppression of protein binding and when a dense brush is achieved (ρ_PEG_ > 0.96 nm^−2^), nonspecific adsorption is nearly eliminated. Figure 2 was created with BioRender. Gao, P. (2025) https://BioRender.com/mpl3g35. This content is not subject to CC BY 4.0.

PEG’s hydrophilic and steric properties are instrumental in reducing the adsorption of proteins onto LNP surfaces. By creating a hydrated barrier, PEG minimizes nonspecific binding, leading to a “stealth” effect that prolongs circulation time and reduces recognition by the mononuclear phagocytic system [24]. However, the effectiveness of this barrier depends on PEG density, molecular weight, and chain configuration. The previously mentioned high-density PEG layers in a “brush” conformation are particularly effective at repelling proteins compared to lower-density “mushroom” configurations, which may permit partial protein adsorption [14–16,25]. The density of engrafted PEG lipids on the LNP surface can be quantified as the number of PEG chains per square nanometer surface area, denoted as ρ_PEG_. As illustrated in Figure 2, serum protein adsorption inversely correlates with ρ_PEG_; low-density regimes, ρ_PEG_ < 0.32 nm^−2^, permit substantial protein binding due to sparse PEG coverage, whereas intermediate densities, 0.32 nm^−2^ < ρ_PEG_ < 0.96 nm^−2^, show partial shielding. High-density PEG layers, ρ_PEG_ > 0.96 nm^−2^, in a “brush” conformation are particularly effective at repelling proteins, achieving nearly complete suppression of nonspecific adsorption [26]. This quantitative relationship underscores that optimizing the PEG density is critical to balance stealth properties with surface interactions in LNP design.

**Figure 3:**
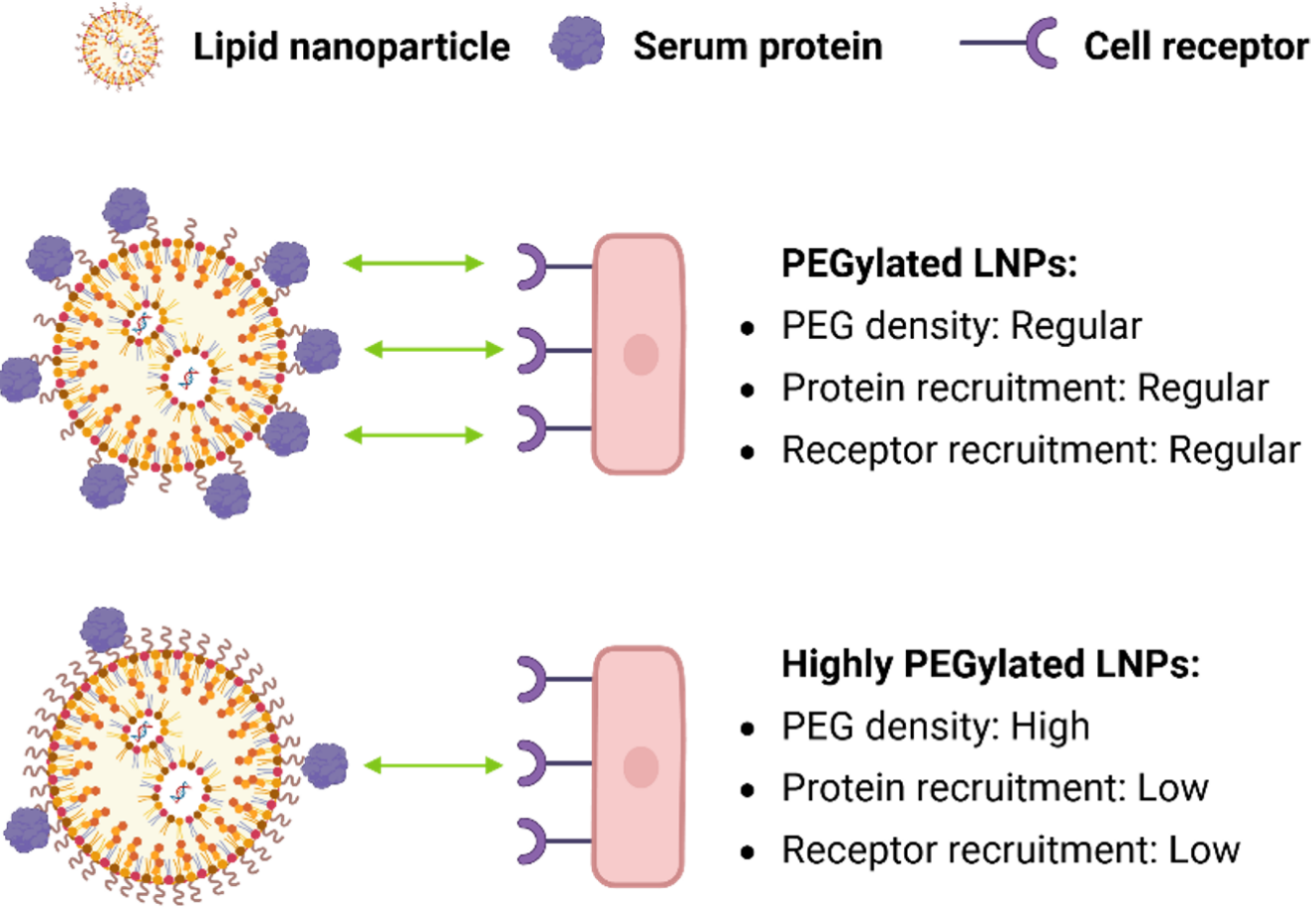
Regular PEGylation density (<3% PEG mol %) retains some accessible LNP surface for protein and receptor recruitment, whereas highly PEGylated LNP surface (>3% PEG mol %) maximizes stealth by markedly reducing both protein adsorption and receptor interactions, ideal for prolonged systemic circulation. This is a representative trade-off between LNP surface stealth and cellular interactions. Figure 3 was created with BioRender. Gao, P. (2025) https://BioRender.com/mpl3g35. This content is not subject to CC BY 4.0.

While increasing PEG density on LNPs can effectively reduce nonspecific protein adsorption, it may also impede the recruitment of functional lipoproteins such as apolipoprotein E (ApoE), which is critical for receptor-mediated hepatic uptake [27–28]. In a systematic study using LNPs containing DMG-PEG2k lipid at different molar fractions of 1.5, 5.0 and 10.0 mol %, both physicochemical characterization and biological performance of LNPs were evaluated. These LNPs were able to maintain comparable sizes in the range of 46 to 58 nm. To further assess the impact of increased PEG density on protein adsorption, ApoE association to LNPs was quantified in vitro using fluorescence-labeled ApoE and size-exclusion chromatography. After 7 min of incubation, strong ApoE binding was observed in the 1.5% PEG LNP, while the 5.0% PEG exhibited significantly reduced ApoE association [20]. As illustrated in Figure 3, the increase in surface PEG density reduces the nonspecific binding of serum protein.

Intravenous administration of these LNPs into C57BL/6 mice further revealed that the LNPs with 1.5% PEG achieved the most potent gene silencing. In contrast, the LNPs with 5.0% and 10.0% PEG required higher doses to achieve similar effects, which demonstrated an inverse relationship between PEG molar fraction and delivery efficiency. Mechanistically, this could be attributed to reduced adsorption of ApoE to the LNP surface, in the case of ApoE acting as an endogenous hepatic targeting ligand [20,28]. A follow-up study using ApoE^−/−^ mice showed that pre-incubation of these LNPs with recombinant ApoE was able to rescue the activity of the LNP with 1.5% PEG but had no effect on the 5.0% PEG counterpart. More importantly, the reduced efficacy observed at higher PEG densities was recovered by incorporating an exogenous targeting ligand. The addition of 0.5 mol % DSG-PEG-GalNAc restored silencing activity in the 5.0% PEG LNP via ASGPR-mediated uptake, functioning independently from ApoE-mediated pathways [20]. Together, these findings point out a critical trade-off: Increasing the molar fraction of PEG enhances immunological stealth but simultaneously impairs delivery through natural LNP uptake pathways. It emphasizes the importance of surface properties and the utilization of possible targeting strategies to enhance cellular uptake [29].

Another research study formulated LNPs with varying molar fractions of DMG-PEG2k from 0.5 to 5.0 mol %, while maintaining a fraction of 50 mol % MC3 in the composition with changing molar fractions of cholesterol or DSPC. Notably, increasing the molar fraction of PEG brought the LNP diameters from 150 nm down to 50 nm, regardless of the molar fractions of DSPC or cholesterol. Subretinal injections into BALB/c and Ai9 reporter mice demonstrated that LNPs with 0.5 mol % PEG elicited, respectively, 18.9-fold and 17.3-fold higher luciferase expression compared to 5 mol % PEG LNPs. Fundus imaging and immunohistochemistry confirmed robust tdTomato fluorescence in the retinal pigment epithelium (RPE), with a twofold to 3.4-fold increase in signal, showing the 0.5% PEG LNPs consistently outperformed 5.0% PEG LNPs [30]. Interestingly, subretinal injected LNPs showed similar expression levels in ApoE^−/−^ and wild-type mice, as well as in Mertk^−/−^ mice lacking RPE phagocytic capacity. While direct in vitro protein corona profiling was not performed, the data collectively suggests that LNPs with lower PEG molar fraction favor cellular uptake and expression, likely by maintaining partial surface accessibility for protein recruitment and receptor-mediated interactions [30–31].

A recent PEG lipid screening study for optimizing LNPs also showed similar results, by testing different PEG lipids at an increased molar fraction from 1% to 5% [32]. In primary cortical neurons treated with 50 nmol antisense oligonucleotide for 24 h, LNPs with 1 mol % PEG achieved up to 80% mRNA knockdown. In contrast, LNPs with 3% and 5% PEG did not reach 50% knockdown even at the highest dosage. This pattern held true across various PEG lipid families and was consistent in serum-containing microglial cultures. Together, these studies consistently demonstrated that increasing PEG density on the LNP surface enhances colloidal stability and reduces nonspecific protein bindings. However, this surface shielding effect on LNPs can also impede cellular uptake in various tissues [29–30,32]. To overcome this trade-off, the use of functionalized PEG lipids may be able to facilitate receptor-specific targeting while maintaining the protection to LNP surface [33].

### Functionalized PEG lipids for ligand conjugation and targeted LNP cellular uptake

**Figure 4:**
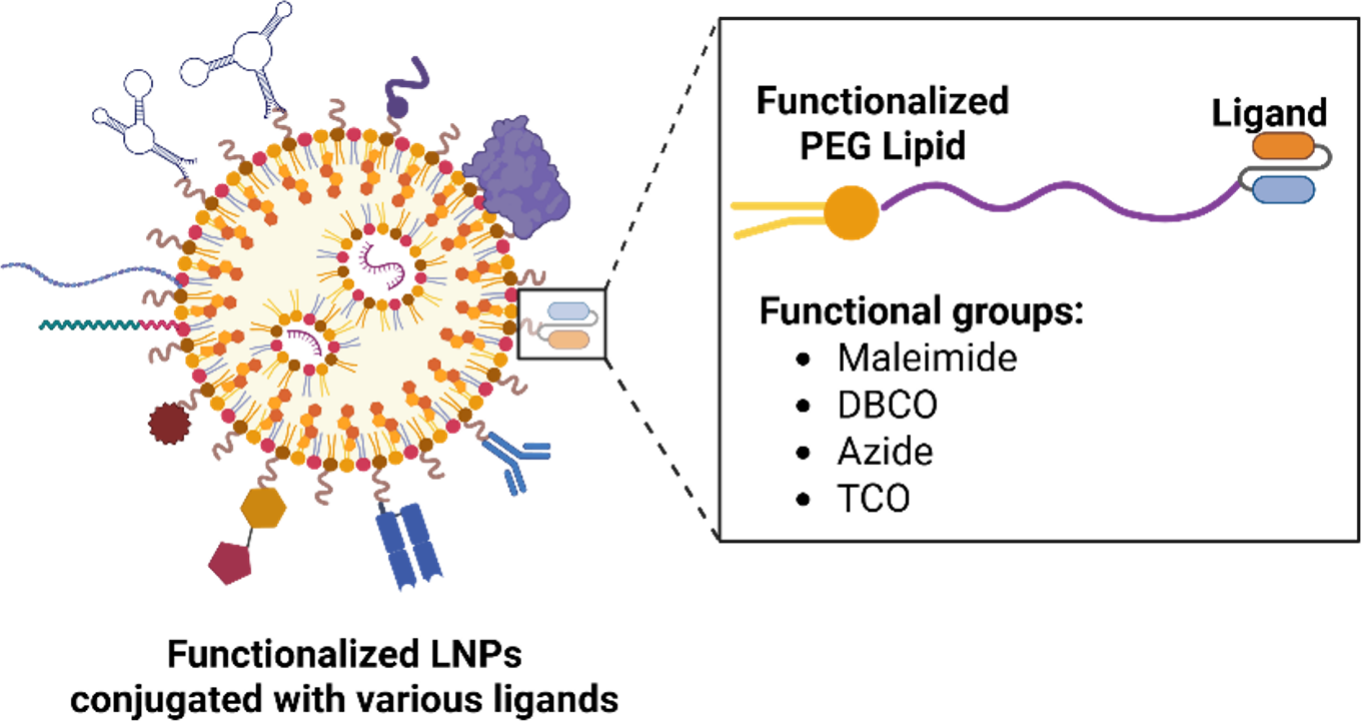
Functionalized PEG lipids incorporated into LNPs to enable surface conjugation of diverse ligands for receptor-mediated targeted delivery. Various functional groups can be incorporated into PEG lipids for ligand conjugations via different complexation mechanisms. Figure 4 was created with BioRender. Gao, P. (2025) https://BioRender.com/mpl3g35. This content is not subject to CC BY 4.0.

To overcome the reduced cellular uptake associated with high PEG density, functionalized PEG lipids can be utilized as chemical handles for the conjugation of small molecules as targeting ligands [34]. For instance, maleimide-PEG lipids are widely used for thiol-mediated conjugation to cysteine residues on antibodies or peptides, enabling site-specific and stable coupling [35]. PEG lipids modified with dibenzocyclooctyne (DBCO) or azide groups can undergo strain-promoted azide-alkyne cycloaddition. This click chemistry is advantageous when conjugating sensitive targeting moieties that might be adversely affected by harsh reaction conditions [36–37]. A selection of functionalized PEG lipids for ligand conjugation is shown in Figure 4.

A study of engineered peptide-functionalized LNPs (pLNPs) used DSPE-PEG-maleimide as a linker to conjugate brain-targeting peptides including RVG29, T7, AP2, and mApoE through thiol-maleimide click chemistry [38–40]. The functionalized PEG lipid was incorporated by substituting 10%, 30%, or 50% of the total 1.5 mol % PEG lipid content with DSPE-PEG-maleimide in the LNP formulation. Peptides containing a terminal cysteine residue were subsequently conjugated to the maleimide groups under mild aqueous reaction. These pLNPs demonstrated substantial enhancements in mRNA transfection in vitro. RVG29 and mApoE pLNPs with 10% DSPE-PEG-maleimide substitution achieved up to 60-fold and 20-fold higher luciferase expression in brain endothelial and neuronal cells than that of the negative control LNP without peptide [40]. Notably, these enhancements remained consistent in pLNPs pretreated with serum, indicating that the peptide-conjugated LNPs using DSPE-PEG-maleimide retained targeting ability despite protein corona formation.

Intravenous injection at 0.3 mg·kg^−1^ further validated the efficacy of these functionalized pLNPs. RVG29 and AP2 pLNPs achieved nearly 70-fold increases in brain transfection, while mApoE pLNPs demonstrated significant tropism shift from liver to spleen. A liver luminescence reduction from over 90% to 25% was observed in negative control versus mApoE pLNPs, with a nearly 160-fold increased spleen-to-liver signal [40]. Fluorescence microscopy further demonstrated that RVG29 pLNPs exhibited enhanced cellular uptake and efficient endosomal escape. These findings support that functionalized PEG lipids containing maleimide groups are effective for ligand attachment to LNP surface and targeted delivery [40–41].

A dual-targeted LNP system composed of two functionalized PEG lipids was created for ligand-mediated targeting of DNA-loaded LNPs to breast cancer cells [42]. DSPE-PEG-folate was directly incorporated into the LNP formulation as a targeting ligand for folate receptor-positive breast cancer cells [43]. The other ligand, anti-HER2 monoclonal antibody Herceptin, was first thiolated using *N*-succinimidyl *S*-acetylthioacetate and then conjugated to DSPE-PEG-maleimide at a 1:4 molar ratio, with unreacted maleimide groups quenched by adding ʟ-cysteine [42,44]. The dual-targeted LNPs exhibited enhanced performance in vitro across breast cancer cell lines including MCF7, MDA-mb453, and SKBR3. In HER2-overexpressing SKBR3 cells, the dual-targeted LNPs achieved 75% transfection efficiency and significantly outperformed single-ligand LNPs with folate or Herceptin only [42]. In vivo validation was performed using a zebrafish larvae xenograft model, in which MCF7 breast cancer cells were implanted into the larvae at 48 h post-fertilization. The LNPs were labeled for tracking and then administered via intravenous injection at 10 ng DNA per dose [42]. Although early tumor accumulation of the dual-targeted LNPs was modest in MCF7 xenografts, confocal microscopy at 45 h post-injection showed enhanced EGFP transgene expression compared to single-ligand LNPs. These findings demonstrated how integrating multiple targeting moieties through functionalized PEG lipids can overcome tumor heterogeneity and enhance cell uptake [42,45].

DBCO-azide click chemistry was used to develop PD-L1-targeted LNPs (Pep-LNPs) for tumor-selective mRNA delivery [46–47]. The functionalized PEG lipid DSPE-PEG2K-DBCO was conjugated with azidoacetyl-modified A PD-L1-binding ᴅ-peptide before introducing to LNP formulation. The synthesized DSPE-PEG2K-Pep conjugate was incorporated into LNPs at 0.3 mol % for optimal coating saturation. Quantitative analysis showed Pep-LNPs achieved over 85% of surface coverage by DSPE-PEG2K-Pep conjugate without sacrificing nanoparticle stability. In vitro results showed that the Pep-LNPs selectively bound to and were internalized by PD-L1 expressing cancer cells including CT26.CL25, HCC1937, and 4T1-Luc, with no enhanced uptake in PD-L1 negative U87MG cells. Pre-treatment with anti-PD-L1 antibody significantly reduced both LNP binding and cellular uptake, confirming that the interaction was mediated specifically through the PD-L1 receptor. Intravenous administration of Pep-LNPs in vivo showed a 4.2-fold increase in tumor accumulation and 2.6-fold higher transgene expression over peptide-free LNPs [47].

Using the same click chemistry, EGFR-targeted LNPs were created in another study to achieve selective mRNA delivery to placental tissue [48–49]. To enable antibody conjugation, DSPE-PEG-azide was incorporated into the LNP formulation by replacing different molar fractions of the total 2.5 mol % PEG content. DBCO-labeled EGFR antibodies were conjugated with the azide-functionalized PEG lipids, and unreacted antibodies were removed by size-exclusion chromatography. The aEGFR-LNPs significantly enhanced luciferase mRNA delivery in EGFR-expressing JEG-3 cells, showing up to threefold increase over controls. Intravenous administration of aEGFR-LNPs at 0.4 mg·kg^−1^ in pregnant mice resulted in a nearly twofold increase in mRNA expression in placentas compared to antibody-free LNPs, without detectable fetal transfer. Flow cytometry also confirmed a twofold increase in aEGFR-LNP uptake in EGFR+ trophoblasts and enhanced accumulation in placental immune cells [49]. These findings further demonstrated that conjugating ligands to functionalized PEG lipids can enhance cellular uptake in targeted LNP delivery [47,49–50].

Interestingly, a recent study showed that, even in the absence of a ligand, functionalized PEG lipids themselves may influence LNP biodistribution [51]. By partially substituting a total of 2.5 mol % DSPE-PEG content with functionalized PEG containing maleimide, carboxylic acid, or PDP, the study systematically examined how the functionalized groups alone can modulate LNP targeting. Maleimide-LNPs showed notably higher cellular association with HepG2 liver cancer cells within 15 min in vitro. Furthermore, after intratumoral injection of maleimide-LNPs at 1 mg·kg^−1^ into HepG2 xenograft-bearing mice, maleimide-LNPs exhibited greater retention at the tumor site compared to non-functionalized LNPs. Imaging and histological analysis showed that maleimide-LNPs remained localized near the injection site, implying enhanced interaction with the tumor microenvironment. In contrast, carboxylic acid-LNPs showed a reduced cellular association, likely due to electrostatic repulsion [52]. PDP-LNPs had minimal effect on uptake or transfection efficiency, suggesting limited interaction enhancement through that functional group. This study highlights that even without ligands, functionalized PEG lipids may be able to modulate LNP surface interactions and biodistribution, though the effect strongly depends on terminal chemistry [51].

Moreover, functionalized PEG lipids can also be used to prolong circulation by adding immune-evasive moieties [53–54]. For instance, CD47 peptides were decorated onto the LNP surface using DSPE-PEG-maleimide with a conjugation efficiency of 89.4% and a surface density of ≈8.9 peptides per nanoparticle. These CD47-LNPs have showed a fivefold increase in blood retention, with a pronounced decrease in liver and spleen accumulation [55]. This suggests that the CD47 addition to LNP surface can engage effectively with SIRPα-expressing phagocytes and reduce clearance [56]. Therefore, functionalized PEG lipids can serve as anchors not only for targeting moieties but also for ligands to reduce immune clearance and extend blood circulation [40,42,47,49,55].

Although functionalized PEG lipids have great potential for LNP surface modifications, recent studies have emphasized the importance of careful handling to mitigate possible safety risk [57–58]. For instance, a DBCO-functionalized PEG lipid was found to induce protein aggregation on the nanoparticle surface, primarily due to the hydrophobic nature of the DBCO group, leading to elevated complement activation. Excessive maleimide can react with thiol-containing plasma proteins and trigger the alternative complement pathway [57]. Therefore, quenching free maleimide groups with cysteine will be required but with added complexity in processing [59]. In addition to these functionalized PEG lipids for click conjugation, trans-cycloctene (TCO) can be considered for its much lower hydrophobicity compared to DBCO [57]. A study using TCO-tetrazine conjugation for ultrasound-guided membrane anchoring highlights the versatility of TCO-functionalized PEG lipids in mediating bioorthogonal conjugation. Successful incorporation of DOPE-PEG-TCO into LNPs and conjugation with tetrazine suggests its potential in targeted LNP delivery [60].

These research findings demonstrated that LNPs functionalized by PEG lipids can significantly enhance cell-specific uptake, supported by both in vitro and in vivo data sets. The incorporation of some functionalized PEG lipids can even improve cell interaction and tumor retention without ligand addition. This strategy represents a practical approach to overcoming the trade-off of conventional PEGylation by maintaining circulation stability while enabling functional interactions with target cells. In cases where higher PEG density is required to maintain LNP stability but may impede cell uptake efficiency, using functionalized PEG lipids can enable LNP surface engineering to achieve both stability and targeted delivery [29,40,51,55].

### Immunogenicity of pegylated LNPs and accelerated blood clearance

Unlike PEG polymers used in pharmaceuticals and consumer products, PEG lipids are chemically bonded to lipid anchors, enabling their incorporation into lipid membranes [61]. PEG polymers are considered biocompatible and have low immunogenicity, supporting their widespread use in many products such as cosmetics, hydrogels, and lubricants [62]. In lipid nanoparticles, PEG is normally incorporated in the form of PEG lipids, which reside in the surface bilayer to reduce particle aggregation and nonspecific binding. However, studies have shown that PEGylated LNPs can elicit immune responses and accelerated blood clearance upon repeated administration [4,63]. This suggests that the immunological behavior of PEG may be altered when attached to a lipid moiety such as DMG or DSPE.

The immunogenicity of PEGylated therapeutics primarily originates from anti-PEG antibodies, which are commonly developed through prior exposure to PEG-containing products [64]. In a recent study, Liu et al. established mouse models with pre-existing anti-PEG antibodies and demonstrated that PEGylated mRNA-LNP vaccines led to diminished spike protein expression with increased complement activation. Such effects were not observed in naive animals, which suggests that the administration of PEGylated LNPs can trigger the recognition by anti-PEG antibodies in sensitized individuals [65]. Another study comparing LNP formulations used in COVID-19 mRNA vaccines revealed distinct immunogenic profiles associated with their PEG lipids [66]. Moderna’s mRNA-1273 vaccine contains approximately 117 μg of PEG lipids in each standard adult injection dosage and significantly increased anti-PEG immunoglobulin G (IgG) and immunoglobulin M (IgM) titers by about 13-fold and 68-fold, respectively. In contrast, Pfizer-BioNTech's BNT162b2 vaccine, with a lower amount of PEG lipid at approximately 50 μg per injection, induced relatively modest increases in anti-PEG IgG and IgM titers at approximately 1.8-fold and 2.6-fold. These findings further demonstrated the immunogenic effect of PEG lipids, particularly at higher dosage levels, even though PEG polymers are broadly considered safe in consumer applications [66–67].

This immunogenicity of PEGylated LNPs not only raises safety concerns but also poses a risk to the structural integrity of the nanoparticles [68]. Anti-PEG antibodies can activate the complement system, leading to the deposition of complement fragments and formation of the membrane attack complex [64]. A study showed that exposure of LNPs containing 5 mol % DSPE-PEG2k to anti-PEG IgM and complement-active serum induced up to 50.5% release of encapsulated mRNA payload. In the widely used LNP composition containing 1.5 mol % DMG-PEG2k, nearly 34% payload leakage was observed as well [68]. These findings imply diminished therapeutic efficacy due to premature payload leakage upon PEGylation-induced immunogenicity.

**Figure 5:**
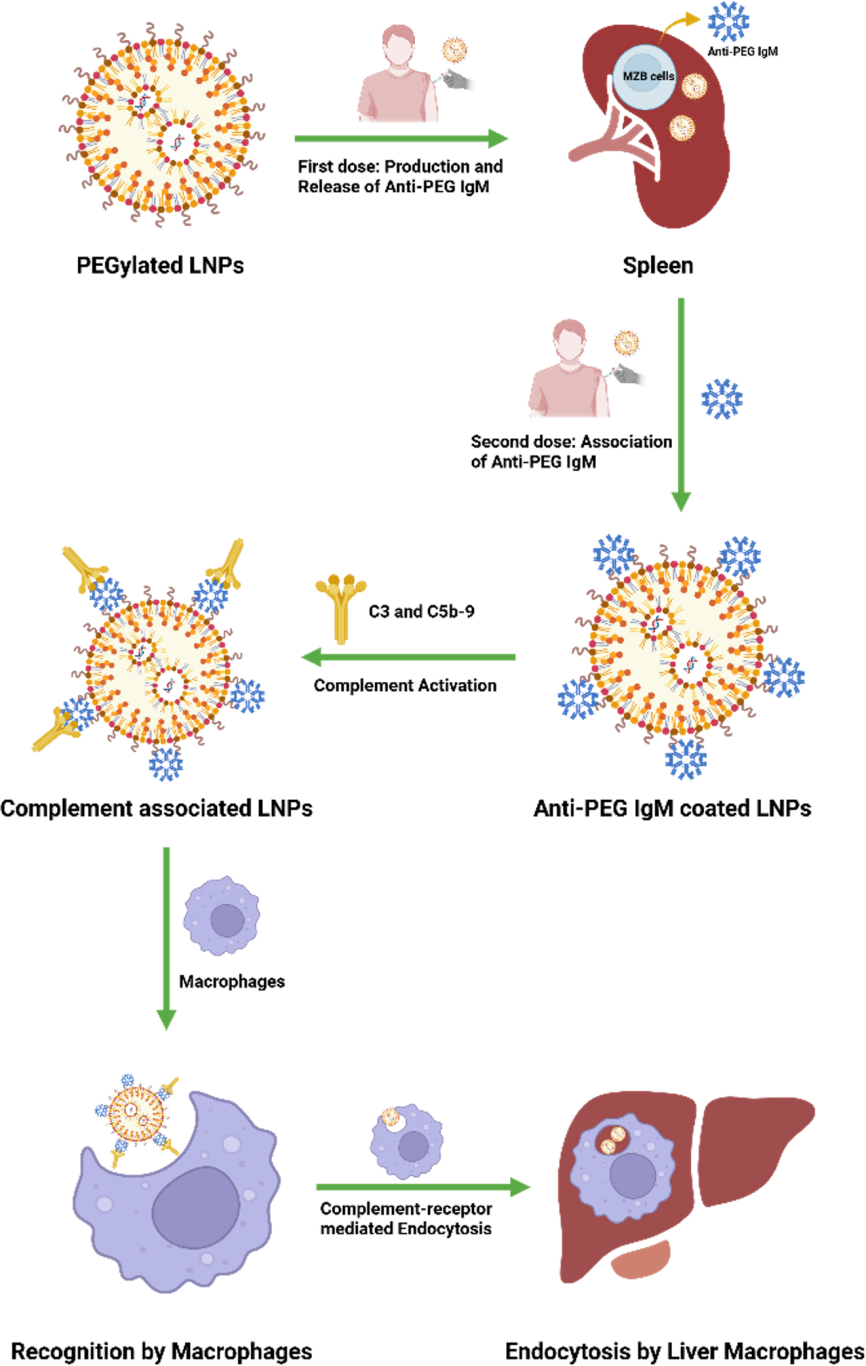
Mechanism of anti-PEG immune response and ABC phenomenon. Initial dosing induces the splenic production of anti-PEG antibodies. Upon subsequent administrations, these antibodies rapidly bind to PEG lipids on the LNPs, triggering the complement pathway as evidenced by C3/C5b-9 deposition and opsonization. This causes LNPs to be recognized and internalized by liver macrophages, resulting in accelerated blood clearance and reduced therapeutic efficacy. Figure 5 was created with BioRender. Gao, P. (2025) https://BioRender.com/mpl3g35. This content is not subject to CC BY 4.0.

Repeated dosing of PEGylated LNPs further amplifies immunogenicity concerns, as the immune system can become primed for a heightened response. As illustrated in Figure 5, anti-PEG antibodies can rapidly neutralize PEGylated nanoparticles, triggering the accelerated blood clearance (ABC) phenomenon [4,69]. This effect is characterized by a dramatic reduction in circulation time for subsequent doses, often leading to diminished therapeutic efficacy and altered biodistribution. Evidence from further animal studies suggests that the ABC effect follows a biphasic timeline, with initial immune priming occurring shortly after the first dose and peak clearance effects observed during subsequent administrations [70]. Complement activation plays a central role in mediating PEG immunogenicity. When anti-PEG antibodies bind to PEGylated LNPs, they can activate the complement cascade via classical pathways. This mechanism leads to the deposition of complement proteins C3 and C5b-9 on the nanoparticle surface, which further enhances immune recognition [69].

An earlier study found that a single dose of PEGylated LNPs can induce anti-PEG IgM responses, with a dosage of 2.35 mg phospholipids kg^−1^ reaching a peak log_10_ IgM concentration of ≈4.26. After a second dose in 21 days, the immunogenic response was significantly amplified, with anti-PEG IgG detected at a log_10_ concentration of ≈2.55. More importantly, serum LNP levels dropped by over 50% compared to the first dose, indicating that anti-PEG antibodies accelerated opsonization and clearance [71]. Interestingly, a recent study found that PEGylated LNPs containing C16-PEG-ceramide triggered markedly higher anti-PEG IgM production at 2279.7 U·mL^−1^, while free C16-PEG-ceramide induced much lower IgM production at 528.3 U·mL^−1^. This implies that the presentation of PEG lipids on LNP surface can be the most significant trigger in immune recognition. Upon repeated dosing, C16-PEG-ceramide LNPs led to elevated complement activation, with C5a levels measured at 348.7 U·mL^−1^ [70]. These findings clearly demonstrated that PEG-associated immunogenicity may compromise LNP therapeutic efficacy, especially upon repeated administration [70–72].

Nevertheless, the immunogenicity of PEGylated LNPs is not without benefit. In vaccine and cancer immunotherapy developments, the innate immune activation triggered by PEG lipids may serve as an adjuvant to promote stronger humoral and cellular responses [73–74]. For instance, PEGylated LNPs were able to induce proinflammatory cytokines such as TNF, IL-6, and IFN-γ; this immune-stimulation supported robust IgG- and IFN-γ-producing T cell responses after repeated dosing [75]. A similar effect was observed in another study, where mice with pre-existing anti-PEG antibodies exhibited a modest increase in OVA-specific IgG levels after mRNA-OVA-LNP vaccination. Specifically, the total IgG absorbance increased in anti-PEG antibody positive mice indicated a ≈29% enhancement compared to control mice [64]. This suggests that the induced anti-PEG antibody production may act as adjuvant to boost the humoral immune response without impairing antigen expression or cytokine induction [64,76].

Collectively, these findings indicate that PEG-associated immune responses can be functionally impactful to PEGylated LNPs. Also, likely unavoidable, conventional PEG lipids and functionalized PEG lipids may both trigger complement activation in different mechanisms [57,65–67]. Although the immunogenicity of PEG lipids can be beneficial in certain cases to modulate immune responses for enhanced therapeutic outcomes, such applications are minimal compared to the well-documented downsides in LNP delivery function. Given this growing body of evidence, while PEG lipids have long been used to maintain LNP stability and reduce nonspecific interactions, their immunogenic liabilities may pose a critical barrier to safe and effective repeated dosing in clinical applications. Therefore, it is important to develop and evaluate alternatives to conventional PEG lipids that retain the key functions of colloidal stability and surface stealth while reducing immune recognition [8,77].

### Alternatives to conventional PEG lipids for enhanced LNP biocompatibility

While PEG lipids have long been the top candidate for enhancing LNP stealth and extending circulation half-life, its limitations such as immunogenicity and accelerated blood clearance have motivated the discovery of alternatives to the commonly used PEG lipids represented by DMG-PEG and ALC-0159 [66–67,71]. In recent years, more promising substitutes to benchmark PEG lipids have been identified. These include structurally distinct polymers with non-PEG backbones and modified PEG derivatives to reduce immune recognition, particularly anti-PEG antibodies [78]. They were evaluated in various LNP compositions to examine the impact on nanoparticle physicochemical characterizations, delivery efficiency, and immunogenicity after partial or complete replacement of original PEG lipids.

Polysarcosine (pSar) is a polypeptoid composed of repeating units of *N*-methylated glycine. It has been explored as a substitute for PEG lipid in LNP formulations to improve the safety and effectiveness of gene delivery [79]. pSar shares many of PEG’s stabilizing properties, such as preventing particle aggregation and providing a hydrophilic barrier, but it offers improved biocompatibility and a lower risk of immune activation [80]. In an earlier study, DMG-PEG lipid was replaced with pSar lipids of different chain lengths and molar fractions in an LNP composition including DODMA, DSPC, and cholesterol [81]. The pSar-LNPs showed particle sizes ranging from 75 to 150 nm varied by the length and molar fraction of pSar incorporated. Their sizes were slightly larger than PEG-LNPs, but payload encapsulation efficiencies were nearly the same. Importantly, in the repeated dosing study of pSar-LNPs, Balb/C mice received weekly intravenous injections for four weeks. Mice treated with pSar-LNPs showed similar or lower levels of liver enzymes compared to those treated with PEG-LNPs, suggesting better hepatic safety. In the same mouse model, strong protein expressions were observed after injection of 10 µg mRNA per dose. Additionally, in vitro studies using human whole blood also revealed that pSar-LNPs triggered lower levels of the proinflammatory cytokines IL-1β, IL-6, and IL-8, compared to PEG-LNPs [81].

Recent studies have further confirmed the potential of pSar to replace PEG lipids in LNP formulations. To evaluate pSar in FDA-approved LNP compositions, PEG lipids were fully substituted with pSar lipids in both ALC-0315- and SM-102 based LNPs at equivalent molar fractions [79]. C57BL/6 mice received intramuscular injections of firefly luciferase or erythropoietin (EPO) mRNA within pSar-LNPs or PEG-LNPs. In ALC-0315 formulations, pSar-LNPs achieved over fivefold higher luminescence and significantly elevated EPO levels in muscle tissue and plasma compared to PEG-LNPs. While SM-102 formulations showed similar protein expression levels between pSar- and PEG-LNPs, the delivery efficiency was at least maintained. Systemic cytokine profiling via Luminex assay showed that pSar-LNPs and PEG-LNPs induced comparable expression of 32 innate immune markers, including CXCL10 and IL-6, with no significant differences observed in most analytes 6 h post-injection. Notably, pSar-LNPs did not induce the production of anti-PEG antibodies after repeated dosing, whereas PEG-LNPs did. Liver enzyme levels remained comparable in both groups, indicating no added hepatic burden. Although enhanced delivery is not guaranteed across all formulations, the reduced immune risk makes pSar a promising candidate for PEG-free LNPs [79,81].

Another study investigated whether pSar can reduce recognition by anti-PEG antibodies. By designing a series of diblock and triblock copolymers, pSar was placed between PEG and a hydrophobic segment to act as a hydrophilic shield [80]. The rationale was that the highly water-soluble and flexible pSar chains could sterically block antibody access to the PEG domain, thereby reducing immune recognition. ELISA showed that the absorbance signal from anti-PEG IgG dropped from over 1.5 to below 0.2 in pSar55 polymer-incorporated LNP. Moreover, this reduced antibody binding was found to be pSar-unit-dependent. It was observed that pSar with 10 and 15 units provided only partial reduction, while the 55-unit pSar showed nearly complete suppression of antibody binding. Although not further validated in vivo, these quantitative results demonstrated that integrating pSar into PEG modifications can significantly reduce anti-PEG antibody recognition.

Poly(2-oxazoline)s are another alternative to PEG lipids with promising results demonstrated by recent LNP studies using poly(2-methyl-2-oxazoline) (PMOZ) and poly(2-ethyl-2-oxazoline) (PEOZ) [82–83]. Structurally, these polymers have short alkyl side chains that contribute to high hydrophilicity and flexibility. Unlike the entangled PEG chains due to interchain hydrogen bonding and van der Waals forces, poly(2-oxazoline) chains have shorter side chains that can prevent strong interchain association. This can create a more uniform and stable hydration shell around the polymers to reduce protein adsorption and prevent complement activation [84]. In LNPs formulated with PMOZ-DM-amide to replace the 1.5 mol % DMG-PEG, payload encapsulation efficiencies were well maintained above 95% and particle sizes were observed within the range between 69 and 80 nm, comparable to PEG-LNPs. BALB/c mice received 1 µg mRNA per dose via intramuscular injection on day 0 and day 21, serum cytokine measurements at 14 h post-injection showed much lower levels of proinflammatory markers in PMOZ-LNPs, including significant reductions in IL-6, MCP-1, and IP-10. Despite slightly lower CD8+ T cell activation, PMOZ-LNPs induced two- to fourfold higher neutralizing antibody titers against the receptor-binding domain compared to PEG-LNPs, as measured by surrogate virus neutralization assays [82]. These results suggest that PMOZ-lipids can reduce systemic immune activation while supporting strong humoral responses.

The analogue PEOZ was evaluated in a more recent study, where the PEG-lipid ALC0159 was replaced with a series of PEOZ lipids of varied chain lengths [83]. Among the PEOZ-LNPs, only PEtOx18 showed notable difference to PEG-LNP control in terms of nanoparticle characterization, with a particle size of 177 nm and an encapsulation efficiency of 77%, compared to the 138 nm size and 86% encapsulation efficiency of PEG-LNP. However, uptake and expression studies using HEK293T cells confirmed that PEtOx18 reached over 90% of cells and sustained strong protein production despite a slight delay compared to PEG-LNP control. These LNPs outperformed PEG-LNP by producing 72% EGFP-positive cells compared to 56% in PEG-LNP. More importantly, the anti-PEG ELISA assay confirmed that none of the PEOZ-LNPs exhibited detectable antibody binding. And after 38 weeks of storage at 4 °C, all nanoparticles were able to maintain their size, encapsulation efficiency, and transfection activity [83]. These data support that PEOZ has the potential to reduce immune activation while maintaining nanoparticle stability.

Another study further validated the potential of PEOZ lipids with promising in vivo results. While the PEOZ lipids used in the two studies differ in anchor structure and linkage chemistry, both share the same poly(2-ethyl-2-oxazoline) backbone [83,85]. In this study, DMG-PEOZ2k and 5k lipids were used to enable direct comparison with DMG-PEG2k. LNP compositions were systematically screened by changing the molar fraction of PEG and PEOZ lipids, with DMG-PEOZ2k at 1.5 mol % identified as the most promising composition. Although the PEOZ2k-LNP exhibited a larger nanoparticle size of 145 nm compared to the 78 nm of PEG-LNP, in vivo studies demonstrated that it achieved equivalent mRNA delivery to liver cells with significantly enhanced transfection in splenic antigen-presenting cells. Additional serum analysis confirmed that anti-PEOZ IgM levels were significantly lower than anti-PEG IgM during the repeated administrations. While it is argued that PEG alternatives may still elicit immune recognition through other pathways, this analysis is insightful to support that DMG-PEOZ2k lipid induces a markedly milder IgM response. Notably, this reduction in anti-polymer IgM was accompanied by improved performance upon repeated dosing, with higher retention of luciferase expression. After four weekly injections, luminescence in the liver dropped by only 67% in PEOZ2k-LNP, compared to 91% in PEG-LNP. Together, these findings suggest that PEOZ is a promising PEG alternative, especially in repeated LNP administration [85].

Another class of PEG alternatives called poly(glutamic acid)-grafted ethylene oxide copolymers (PGE) were evaluated and expected to address the anti-polymer antibody concern. A key advantage of PGE is attributed to its short oligoethylene oxide side chains, each in the range of 2–5 units [86]. Structural studies of anti-PEG antibodies have shown that effective binding requires a stretch of at least seven ethylene oxide units [87]. It implies that this short PGE chain of PGE should be able to reduce the antibody binding affinity. More importantly, such short length is also unlikely to induce anti-polymer antibody generation due to insufficient epitope length for B-cell receptor clustering and activation [88]. In addition, the poly(glutamic acid) backbone, known for its low immunogenicity even when conjugated to protein, can further minimize the risk of immune priming [89]. Together, these make PGE promising especially in evading anti-polymer antibodies, which may impede other PEG alternatives.

To verify the potential of PGE as more potent PEG alternatives, PGEs with ethylene-oxide side chains of 2–5 units were synthesized and conjugated to cholesterol [86]. Among these PEG variants with different unit lengths, PGE_3_ formulated into PGE_3_-LNPs at 0.84 mol % exhibited the best performance. In terms of nanoparticle characterization, PGE_3_-LNPs exhibited a markedly smaller size at 50 nm, which is nearly half the diameter of PEG-LNPs. Despite this difference, PGE_3_-LNPs achieved comparable transgene expression, with luciferase activity levels in both liver and muscle tissues equivalent to those of PEG-LNPs after intravenous and intramuscular administration. After three weekly injections of hEPO mRNA at 0.05 mg·kg^−1^, PEG-LNPs showed a dramatic loss of delivery efficacy, with serum hEPO protein levels dropping by over 90%. In contrast, PGE_3_-LNPs were able to maintain over 60% of protein expression after repeated dosing. Furthermore, ELISA analysis showed no detectable anti-PEG antibodies in the mice serum treated with PGE_3_-LNPs [86]. While anti-polymer antibody levels were not directly measured, the sustained delivery efficacy and absence of anti-PEG antibodies support the design rationale that PGE polymers are effective in minimizing immunogenicity.

Poly(*N*-methyl-*N*-vinylacetamide) (PNMV) appears to be another promising alternative to PEG lipids as evaluated in LNP formulations. Its hydrophilic and flexible polyvinylamide backbone is believed to reduce immune recognition [90]. An in vitro study showed that LNPs formulated with DSPE-PNMVA24 to replace DSPE-PEG achieved over 65% GFP knockdown, which outperformed DSPE-PEG control with 40% knockdown. In vivo immunogenicity analysis demonstrated that PNMVA24-LNP was able to maintain prolonged circulation and induced less anti-IgG without further anti-IgM responses after repeated injection. In contrast, PEG-LNP showed a threefold IgG increase and elevated IgM. On top of that, PNMVA24-LNP achieved over 80% gene silencing in tumor cells, whereas PEG-LNP control was below 50% [91].

Linear polyglycerol (lPG) features a polyether backbone with hydroxy groups that reduce hydrophobic interactions and antibody binding. It was evaluated by replacing DMG-PEG2k in mRNA-loaded LNP formulations [92]. In a study, multiple lPG polymers with varied length were incorporated into nanoparticles, and the best-performing lPG was the one having a chain length similar to that of PEG2k. This lPG-LNP had a particle size of 223 nm, which was much larger than the size of PEG-LNP at 110 nm. Regardless of the size difference, lPG-LNP yielded over 43% eGFP-expressing HepG2 cells, with fluorescence intensity comparable to PEG-LNPs and cell viability remaining above 90%. Moreover, lPG-LNP showed negligible anti-PEG antibody binding at approximately 0.83 ng/mL, whereas PEG-LNP exhibited significantly higher binding at 1300 ng·mL^−1^ [92]. These in vitro results demonstrated the potential of lPG to be used as a PEG alternative, but substantial differences in LNP characterization were observed, and further in vivo validation will be needed.

Hydroxy-terminated PEG (OH-PEG) differs from methoxy-terminated PEG by its functional end group, offering a potential strategy to modulate antibody recognition. Commonly used PEG lipids such as DMG-PEG and DSPE-PEG have methoxy as their terminal capping group. This methoxy group is chemically stable and contributes to hydrophilicity. However, it is also the primary epitope recognized by pre-existing anti-PEG antibodies. Studies have shown that the immune antibodies, particularly IgM and IgG, specifically target the methoxy terminus of PEG [93]. Therefore, OH-PEG was noticed for its hydroxy end group, which is not a known target of anti-PEG antibodies. This structural uniqueness of OH-PEG may reduce immunogenicity by minimizing antibody recognition. In a study using OH-PEG to replace PEG with methoxy groups (MeO-PEG), the OH-PEG-LNPs demonstrated over 90% reduction in antibody binding compared to MeO-PEG-LNPs. Isothermal titration calorimetry further confirmed that there was negligible binding between OH-PEG and pre-existing anti-PEG antibodies. After incubation in anti-PEG antibody-positive human serum, OH-PEG-LNPs showed minimal complement activation with significantly lower C3 cleavage and C5a levels, while MeO-PEG-LNPs showed pronounced complement response. Moreover, OH-PEG-LNPs preserved over 95% of mRNA encapsulation after 2 h of incubation, whereas MeO-PEG-LNPs lost nearly 50% of payload [94]. These results collectively highlight the potential of OH-PEG in reducing LNP immunogenicity, though in vivo validation is needed to confirm translatability.

As mentioned before, the activation of antigen-specific immune responses can be beneficial to therapeutic success, particularly in the context of vaccine development. This makes polysorbate-80 (PS-80) a distinctive alternative to PEG lipids. PS-80 is a branched PEG-like surfactant with a hydrophilic polyethylene oxide chain and oleic acid-based hydrophobic tail [95]. It has been already approved for use in licensed vaccine products. To assess PS-80’s potential in replacing PEG lipids in LNPs, a study was conducted to formulate mRNA-LNPs using PS-80 and compare with LNPs containing DMG-PEG2k [96]. In murine models, PS-80 LNPs exhibited three- to fivefold higher expression in the spleen compared to PEG-LNPs after intramuscular injection, with spleen-to-liver signal ratios exceeding 50. Furthermore, PS-80 LNPs encapsulating SARS-CoV-2 spike mRNA induced high anti-spike IgG titers and strong pseudovirus neutralization after a 5 µg intramuscular single dose. These findings indicated the capability of PS-80 LNPs in shifting biodistribution toward immune-relevant organs such as spleen. Also, PS-80 LNPs can leverage immunogenicity to enhance vaccine efficacy by facilitating antigen-specific responses rather than immune clearance issue associated with conventional PEG lipids [95–96].

## Conclusion

PEG lipids have played a significant role in the development and success of LNP-mediated drug delivery. Their hydrophilic coating of nanoparticle surfaces can help maintain colloidal stability and reduce nonspecific interactions during blood circulation [4,9]. The structural features of PEG lipids including chain length, molecular weight, and lipid anchor are key parameters in modulating the physicochemical properties and biological behavior of LNPs [17–18]. Increased PEG surface density can facilitate immune evasion; however, it may suppress cellular uptake and hinder endosomal escape [20–21,27]. In addition, repeated administration of PEGylated LNPs can induce anti-PEG antibody production and then trigger complement activation [70]. This can lead to accelerated blood clearance and, thereby, reduce the therapeutic efficacy of drug-loaded LNPs [72]. These trade-offs represent the difficulty of finding a balance to maintain both LNP stealth effect and delivery efficiency in the use of conventional PEG lipids.

To address these limitations, functionalized PEG lipids were developed for enhancing cell-specific delivery and improving LNP performance [15]. Some of these functionalized PEG lipids are equipped with reactive groups such as maleimide, DBCO or azide, which allow for efficient conjugation of targeting ligands such as antibodies and peptides [40,42,49]. Others may incorporate targeting moieties directly into the PEG structure, such as PEG-folate lipid, which can interact directly with cell receptors [42–43]. The utilization of these functionalized PEG lipids enables the LNPs to maintain stealthiness during circulation while improving selective cellular uptake.

In addition, polymers such as pSar, PEOZ, and PGE have appeared to be promising alternatives to conventional PEG lipids [79,82,92]. These polymers have been evaluated as PEG substitutes in LNP formulations and demonstrated comparable or improved delivery efficacy with reduced immune activations. Other PEG-like alternatives such as OH-PEG and PS-80 have demonstrated features that make them suitable for applications in immunotherapy and vaccine development [94,96]. Despite encouraging findings, most of these alternatives have only been tested in limited contexts. Their promising performance could be LNP composition-specific or depend on administration routes. In addition, several alternatives have shown notable impacts on LNP physicochemical properties, particularly nanoparticle diameter [85–86,92]. Although their delivery performance and reduced immunogenicity are well supported, these differences in nanoparticle characteristics may require additional optimization. Moreover, broader validations across diverse therapeutic cargos, disease models, and dosing regimens will be needed to fully reveal their potential in the clinical application of LNP products.

Moving forward, the advancement of LNPs can utilize surface engineering strategies that enhance cell-specific uptake while maintaining nanoparticle stability and prolonging circulation half-life. Achieving this balance will require a deeper understanding of how the functionalized PEG lipids and ligand decorations may influence LNP behavior in biological environments [97]. Continued development of PEG alternatives and PEG-free LNP platforms can be another practical approach to overcome immune-related limitations and improve delivery efficiency [8]. Together, these efforts will support the creation of more effective and biocompatible LNP-based drug products.

## Data Availability

Data sharing is not applicable as no new data was generated or analyzed in this study.
